# Prevalence of malaria in pregnancy in southern Laos: a cross-sectional survey

**DOI:** 10.1186/s12936-016-1492-2

**Published:** 2016-08-26

**Authors:** Valérie Briand, Jean-Yves Le Hesran, Mayfong Mayxay, Paul N. Newton, Gwladys Bertin, Sandrine Houzé, Sommay Keomany, Yom Inthavong, Nanthasane Vannavong, Keobouphaphone Chindavongsa, Bouasy Hongvanthong, Nadine Fievet

**Affiliations:** 1 Faculté de Pharmacie, Institut de Recherche pour le Développement (IRD), Mère et enfant face aux infections tropicales (UMR216), 4 avenue de l’Observatoire, 75006 Paris, France; 2 COMUE Sorbonne Paris Cité, Faculté de Pharmacie, Université Paris Descartes, Paris, France; 3 Lao-Oxford-Mahosot Hospital-Wellcome Trust Research Unit (LOMWRU), Microbiology Laboratory, Vientiane, Lao People’s Democratic Republic; 4 Faculty of Postgraduate Studies, University of Health Sciences, Vientiane, Lao People’s Democratic Republic; 5 Centre for Tropical Medicine and Global Health, Nuffield Department of Clinical Medicine, University of Oxford, Oxford, UK; 6 Laboratoire de Parasitologie, CNR du Paludisme, AP-HP, Hôpital Bichat, Paris, France; 7 Salavan Provincial Hospital, Salavan, Salavan Province Lao People’s Democratic Republic; 8 Vapi District Hospital, Vapi, Salavan Province Lao People’s Democratic Republic; 9 Champasack Provincial Health Office, Champasack, Champasack Province Lao People’s Democratic Republic; 10 Center of Malariology, Parasitology and Entomology, Vientiane, Lao People’s Democratic Republic

**Keywords:** Malaria, Pregnancy, Low birth weight, Epidemiology, Molecular biology, Laos

## Abstract

**Background:**

There are no data on the burden of malaria in pregnancy (MiP) in Laos, where malaria still remains prevalent in the south.

**Methods:**

Two cross-sectional surveys were conducted in 2014 to assess the prevalence of MiP in Vapi District, Salavan Province, southern Laos: the first consisted of screening 204 pregnant women during pregnancies [mean (95 % CI) gestational age: 23 (22–25) weeks] living in 30 randomly selected villages in Vapi District; the second was conducted among 331 pregnant women, who delivered during the study period in Vapi and Toumlane District Hospitals and in Salavan Provincial Hospital. Peripheral and placental malaria was detected using rapid diagnostic tests (RDT), thick blood smears (TBS) and real-time quantitative polymerase chain reactions (RT-qPCR). Factors associated with low birth weight (LBW) and maternal anaemia were assessed.

**Results:**

In the villages, 12/204 women (5.9 %; 95 % CI 3.1–10.0) were infected with malaria as determined by RT-qPCR: 11 were *Plasmodium vivax* infections and 1 was mixed *Plasmodium vivax*/*Plasmodium falciparum* infection, among which 9 were sub-microscopic (as not detected by TBS). History of malaria during current pregnancy tended to be associated with a higher risk of MiP (aIRR 3.05; 95 % CI 0.94–9.88). At delivery, two *Plasmodium falciparum* sub-microscopic infections (one peripheral and one placental) were detected (4.5 %; 0.6–15.5) in Vapi District. In both surveys, all infected women stated they had slept under a bed net the night before the survey, and 86 % went to the forest for food-finding 1 week before the survey in median. The majority of infections (94 %) were asymptomatic and half of them were associated with anaemia. Overall, 24 % of women had LBW newborns. Factors associated with a higher risk of LBW were tobacco use (aIRR 2.43; 95 % CI 1.64–3.60) and pre-term delivery (aIRR 3.17; 95 % CI 2.19–4.57). Factors associated with a higher risk of maternal anaemia were no iron supplementation during pregnancy, Lao Theung ethnicity and place of living.

**Conclusions:**

The prevalence of MiP in this population was noticeable. Most infections were asymptomatic and sub-microscopic vivax malaria, which raises the question of reliability of recommended national strategies for the screening and prevention of MiP in Laos.

**Electronic supplementary material:**

The online version of this article (doi:10.1186/s12936-016-1492-2) contains supplementary material, which is available to authorized users.

## Background

In 2007, an estimated 125 million women became pregnant in areas of malaria transmission. Of them 62 %, corresponding to 77 million women, were living in the Asia–Pacific region where malaria transmission is low or unstable [1]. Malaria in pregnancy (MiP) is responsible for poor maternal and child outcomes, including maternal anaemia, low birth weight (LBW), due to intra-uterine growth retardation and pre-term birth, which are important contributors to infant morbidity and mortality [2]. In these settings, MiP is also associated with spontaneous abortion, stillbirth and maternal death [3]. A single infection in pregnancy, even asymptomatic or sub-microscopic, can be detrimental to a mother and her fetus, in particular in early pregnancy, when most of the women do not attend antenatal care (ANC) [3]. Few studies have reported on the prevalence and consequences of MiP in low or unstable malaria transmission areas, and particularly in Southeast Asia, compared to high transmission areas [3]. In a recent review, the proportion of women with parasitaemia during pregnancy in the Asia–Pacific region has been estimated to be 15 % (range 1.2–40.8) based on cross-sectional surveys, and as high as 36.5 % (range 6.0–64.0) based on longitudinal studies [3].

In Laos, malaria is endemic throughout most of the country, but intensity of transmission is variable, with more intense transmission in remote and forested areas particularly in the south [4]. The distribution of insecticide-treated nets (ITNs) at household level, free screening and case management of clinical malarial infections and the large-scale distribution of artemisinin-based combination therapy (ACT), have contributed to reducing the incidence of malaria. However, clinical malaria still remains a serious public health problem in the five southern provinces of the country, where a high number of clinical cases are still reported in ‘hot spots’ of malaria transmission (Centre of Malariology, Parasitology and Entomology, CMPE Malaria Information System, unpublished observations). Most patients are adults living in villages who work in forest-related occupations or who are migrating adults workers, sometime accompanied by their families, according to work availability [5]. In Laos, malaria screening for pregnant women is only performed when they present with fever. Therefore, asymptomatic and sub-microscopic infections as well as symptomatic women who do not access health services are neither detected nor reported. The lack of precise information on the burden of MiP in Southeast Asia, and particularly in Laos, has hampered effective lobbying for the inclusion of malaria preventive strategies during pregnancy [6]. In Laos, interventions against malaria that are recommended during pregnancy are the same as for the general population, they consist of use of ITNs and effective treatment of symptomatic malaria [7].

In the context of persistent malaria transmission in southern Laos, the prevalence of MiP, including asymptomatic and sub-microscopic infection, and its related morbidity in this population were assessed. Epidemiological methods that minimize selection bias as well as highly sensitive methods of detection for malaria were used.

## Methods

The study was conducted in Salavan Province, which is one of the five southern provinces where the reported number of clinical malaria cases has been the highest since 2011 (CMPE Malaria Information System, unpublished observations). In this area, malaria is perennial with one peak during the rainy season, which occurs between May and October.

**Fig. 1:**
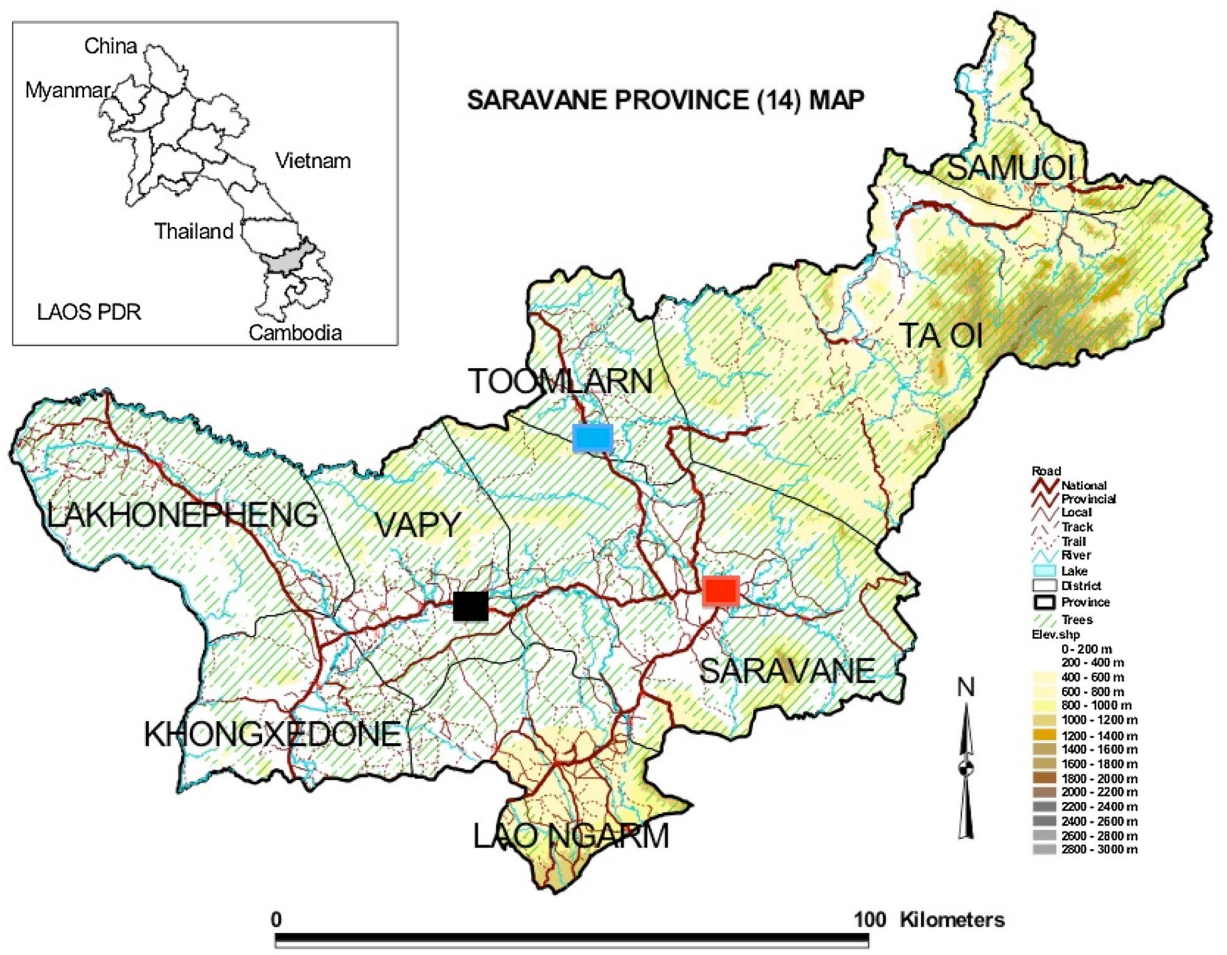
Study sites, Salavan Province, Laos, 2014. The *blue box* corresponds to Toumlane District Hospital, the *red box* to Salavan Provincial Hospital and the *black box* to Vapi District Hospital. Source: World Health Organization, Lao PDR

Two cross-sectional surveys were carried out simultaneously. One was conducted at facility level to investigate MiP at delivery, at Salavan Provincial Hospital (latitude 15.817107 N, longitude 106.252214 E, altitude 183 masl), which is a primary-secondary hospital, and in Vapi (latitude 15.66526 N, longitude 105.906837 E, altitude 136 masl) and Toumlane (latitude 15.993951 N, longitude 106.191913 E, altitude 197 masl) District Hospitals, which are located in more rural areas (Fig. 1).

The second survey was conducted at community level in the villages of Vapi District as a one-time point survey of pregnant women. Data were collected during the rainy season (i.e., malaria transmission period) from July to October 2014. Both surveys were part of a larger programme (PALULAO, FEI n°13INI144), which aimed to assess the prevalence of asymptomatic and sub-microscopic malarial infections in the general population, including pregnant women, adults and children. This programme was led by the CMPE in Laos, in collaboration with the Salavan Provincial Department of Health, the Research Institute for Development (IRD) and the Lao-Oxford-Mahosot Hospital Wellcome Trust Research Unit (LOMWRU).

### Study populations and designs

At facility level, all women who delivered in one of the three participating maternity clinics between August and October 2014 were included after providing a written informed consent. Women were excluded from the study if they had abortion, complicated/at risk pregnancy requiring a pre-labour emergency or elective caesarean section. The following information was recorded on admission by specifically trained midwives: maternal characteristics (age, ethnicity, place of living, education level), obstetrical history (gravidity, number of spontaneous abortions, stillbirths, and live births), clinical data related to the current pregnancy (number and place of ANC visits, obstetrical complication, iron and folic acid supplementation, tobacco use, malaria history, and bed net use the night before admission), and working in the forest during the current pregnancy. At delivery, gestational age, blood pressure, temperature, and delivery characteristics were recorded. Newborn anthropometric data (weight, height and head circumference), gender, Apgar score and congenital abnormalities were recorded. Newborn weight was measured within 1 h after birth using an electronic digital scale with an accuracy of 100 g (SECA Ltd). In addition, maternal peripheral, placental and umbilical cord blood samples were drawn for malaria diagnosis using rapid diagnostic tests (RDT), thick and thin blood smears (TBS) and real-time quantitative polymerase chain reactions (RT-qPCR). Haemoglobin (Hb) level, and proteinuria and glycosuria [using a urine dipstick test (Cybow 2GP^®^)] were determined.

At the community level, the cross-sectional survey was carried out in 30 randomly selected villages out of a total of 53 villages in Vapi District. The day before the survey the head of the village made an announcement inviting all pregnant women to participate in the study. Moreover, women with suspicion of pregnancy were encouraged to visit the study team, which performed a urinary pregnancy test. Maternal characteristics, obstetrical history, clinical data related to the current pregnancy (including bed net use the night before the survey) and activities in the forest were collected. Women were screened for malaria using RDT, TBS and PCR whether or not they were symptomatic. Hb level and proteinuria and glycosuria were determined.

In both surveys, data quality was monitored by study coordinators on a weekly basis. Women with positive RDTs for malaria as well as anaemic women were treated according to the national guidelines.

### Laboratory investigations

RDT (Malaria antigen Pf/Pv, SD Bioline 05FK80, Europ Continents Ltd) was performed on all peripheral blood samples. Blood collected on filter papers was dried and conserved at room temperature until DNA extraction using Chelex [8]. RT-qPCR was performed on peripheral, placental and umbilical cord blood samples for sub-microscopic infection detection. A duplex real-time PCR assay, using genus-specific and species-specific primers and probes for gene encoding the small sub-unit (18S) of *Plasmodium* rRNA, as described by Diallo et al. [9], was used. Samples underwent 40 cycles of amplification using the ViiA™ 7 real-time PCR system (Applied Biosystem) and were quantified using a DNA standard range made from a suspension of in vitro culture of the 3D7 *P. falciparum* line (MR4). PCR were performed in the Microbiology Laboratory of Mahosot Hospital, Vientiane, Laos. A quality control for PCR was performed in Hôpital Bichat, France. DNA extracts of 30 % randomly selected PCR-positive samples and 13 % randomly selected PCR-negative samples were analysed in blind with the FTD (Fast-Track Diagnostics) Malaria PCR, a multiplex PCR for detection of *Plasmodium* spp. DNA. In case of positive result, the PCR FTD Malaria differentiation was performed to identify the species of *Plasmodium*. Agreement between both methods for PCR was high (Cohen’s kappa coefficient: 0.92). Agreement was complete for the 48 samples from pregnant women which were analysed.

TBSs were stained with Giemsa. All placental blood smears as well as a sub-sample of peripheral blood smears (including all PCR-positive samples) were read, and parasitaemia was quantified by the Lambaréné method [10]. TBS reading was performed on site by CMPE staff and Salavan Provincial Hospital staff. Infections were considered sub-microscopic if malaria parasites were detected by qPCR but absent by microscopy. Hb level was determined by HemoCue^®^ (community-level survey) or coulter counter (HUMACOUNT 60TS) (facility-level survey).

### Statistical analysis

Maternal characteristics were described using proportions (95 % confidence interval) for categorical variables and means (95 % CI) and medians (IQR) for continuous variables. The proportion and its exact Poisson 95 % CI of women infected with malaria using RDT and PCR were calculated.

Maternal characteristics associated with malaria during pregnancy (community-level study) were assessed using a modified Poisson regression model [11]. For each covariate, the incidence rate ratio (IRR) and its 95 % CI were estimated. Factors associated with malaria at delivery and its related maternal and child morbidity (facility-level study) could not be assessed because of the low number of malarial infections. However, maternal sociodemographic and obstetrical characteristics associated with low birth weight (LBW) and maternal anaemia at delivery were assessed. Maternal anaemia was defined as a haemoglobin level less than 11 g/dL. Mean Hb level at delivery, mean birth weight and small-for-gestational age (SGA)—as a proxy for intra-uterine growth restriction—were considered as secondary outcomes. SGA was defined as a birth weight below the 10th percentile of the birth weight-for-gestational age according to INTERGROWTH charts [12]. Gestational age and how it had been determined (ultrasound scan, date of last menstrual period (LMP) or fundal height measurement) was recorded from the maternity clinic registers. Twins and stillbirths were excluded from birth weight and SGA analyses. For each outcome, univariate analyses were first performed using linear regression and modified Poisson models depending on the outcome analyzed. Variables for which P value was less or equal to 0.25 were further included in a subsequent multivariate model. The variables were then introduced together in a multivariate regression and were eliminated step by step using the backward method. At this level, only variables for which the P value was less than 0.05 were kept. We considered the following covariates: ethnicity, place of living, maternal age (transformed into a four class variable corresponding to the quartiles), gravidity (primigravidae or multigravidae), tobacco use, forest activity during the current pregnancy, gestational hypertension, use of bed net the night before admission, number of ANC visits (zero, one–three, ≥four visits) iron and folic acid supplementation during the current pregnancy (intake the day before admission, intake but not the day before admission, no intake), maternal anaemia at delivery (for birth weight analyses only), duration of pregnancy (<37, 37–38, ≥39 weeks of gestation) and sex of the newborn. Bed net use was forced in all multivariate models.

Data entry and analysis were performed using Epidata (version 3.1) and Stata (version 13), respectively.

## Results

### Community survey

At community-level, 205 pregnant women (median (IQR) of six women (five) per village) were included (Table 1). Women were primi-, secondi- and multigravidae in 28.8, 30.7 and 40.5 % of cases, respectively. Mean (95 % CI) gestational age was 23 (22–25) weeks as reported by women. It had been determined from LMP (67.5 %), ultrasound scan (20.2 %) or by fundal height measurement (12.3 %). Gestational hypertension without proteinuria was detected in only one woman. History of malaria during pregnancy was reported by 8.3 % of women. Only two women out of a total of 205 (1.0 %; 95 % CI 0.1–3.5) were infected with malaria using RDTs and these two persons were asymptomatic. Malaria was detected using RT-qPCR in 204/205 women, for one woman venous blood was not available. Twelve women out of a total of 204 (5.9 %; 95 % CI 3.0–10.3) were infected with malaria using PCR. Nine (9/12, 75 %) of these infections were sub-microscopic (as not detected by microscopy). Eleven of these infections were vivax malaria, and one was mixed *P. vivax/P. falciparum* infection (Table 2). All infected women were asymptomatic, except for one woman who had history of fever the day before the survey. All women except one stated that they slept under a bed net the night before the survey. Ninety-two percent of infected women (vs 82 % of uninfected women) had been to the forest within the previous 2 weeks to find food.

**Table 1:** Maternal characteristics during pregnancy, Salavan Province, Laos, 2014

	Community survey N = 205	Hospital survey N = 331
	n (%) or mean (95 % CI)	
Maternal age (years)	24.9 (24.0–25.7)	25.0 (24.4–25.7)
Gravidity		
G1–G2	122 (59.5)	220 (66.5)
G3+	83 (40.5)	111 (33.5)
Ethnicity		
Lao Loum	204 (99.5)	266 (80.4)
Lao Theung	1 (0.5)	65 (19.6)
Place of living/delivery		
Vapi	205 (100.0)	44 (13.3)
Toumlan		40 (11.8)
Salavan		248 (74.9)
Education^a^		NA
No formal education	25 (12.8)	
Primary school	103 (52.5)	
Secondary school or higher	68 (34.7)	
Number of ANC visits during this pregnancy	1.4 (1.1–1.6)	3.8 (3.6–4.1)
Iron supplementation^a^	103 (50.2)	310/330 (93.9)
Folic acid supplementation^a^	0 (100.0)	258/327 (78.9)
Gestational hypertension^a^	1/204 (0.5)	11 (3.3)
Tobacco use	NA	38 (11.5)
Went to the forest during the current pregnancy	170 (82.9)	141 (42.6)
Bed net possession	204 (99.5)	307 (92.8)
Bed net use the night before the survey/admission	204 (99.5)	305 (92.1)
Any malaria episode during pregnancy^a^	17/204 (8.3)	7/330 (2.1)
Gestational age (weeks of gestation)^a^	23 (22–25)	38 (38-38)
Maternal Hb level (g/dL)^a^	10.8 (10.6–11.0)	11.4 (11.3–11.6)
Maternal anaemia (Hb < 11 g/dL)^a^	106 (51.7)	112/327 (34.3)

*ANC* antenatal care, *Hb* haemoglobin

^a^N = 331 women at delivery, N = 205 during pregnancy, or indicated otherwise. Blood pressure was not recorded for one woman (community-level survey), history of malaria could not be determined in two women (one during the community-level survey and one during the facility-level survey), education level was not recorded in nine women who were recruited in the first three investigated villages in Vapi District, iron and folic acid supplementation could not been determined in, respectively, one and four women during the facility-level survey, and Hb level could not be measured in four women in Toumlane District Hospital because of a defective device

**Table 2:** Prevalence of peripheral malaria in pregnancy, Salavan Province, Laos, 2014

		Number tested	n (%) RDT+	n (%) PCR+			
				*Pf*+	*Pf* + *Pv*+	*Pv*+	Total
Community survey (during pregnancy)	Vapi	205	2 (1.0)	0 (0.0)	1 (0.5)	11 (5.4)	12/204 (5.9)^a^
Hospital survey (at delivery)	Vapi	44	0 (0.0)	2 (4.5)	0 (0.0)	0 (0.0)	2 (4.5)
	Salavan	248	0 (0.0)	0 (0.0)	0 (0.0)	0 (0.0)	0 (0.0)
	Toumlane	39	0 (0.0)	0 (0.0)	0 (0.0)	0 (0.0)	0 (0.0)

*Pv P. vivax*, *Pf P. falciparum*

^a^RT-qPCR was performed in 204/205 women (venous blood was not available for one woman)

In univariate analysis factors significantly associated with MiP were history of malaria during the current pregnancy and a low education level. In multivariate analysis only history of malaria remained associated with MiP, although this association was only marginally significant (aIRR 3.05; 95 % CI 0.94–9.88). The prevalence of maternal anaemia was 51.7 % (106/205) with overall mean (95 % CI) Hb of 10.8 (10.6–11.0) g/dL (Table 1).

### Hospital survey

A total of 331 pregnant women were included at delivery: 248 (74.9 %) in Salavan, 44 (13.3 %) in Vapi and 39 (11.8 %) in Toumlane Hospitals (Table 1). Women were primi-, secondi- and multigravidae in 38.4, 28.1 and 33.5 % of cases, respectively. Their mean (95 % CI) frequency of ANC visits was 3.8 (3.6–4.1) during their pregnancies. Gestational age had been determined using ultrasound scan in 84 % of women, from the LMP in 3.6 % and by fundal height measurement in 12.4 %. Gestational hypertension was detected in 3.3 % of women with no pre-eclampsia. Glycosuria was detected in only two women, for whom no further biological tests could be performed to confirm gestational diabetes. History of malaria during current pregnancy was reported by 2.1 % of women. Bed net use the night before admission was reported by 92.1 % of women. Looking for food in the forest during current pregnancy was reported by 42.6 % of women. The median (IQR) days of the last stay in the forest before delivery was seven (24). At delivery, sub-microscopic malarial infections were detected in 2/44 (4.5 %; 95 % CI 0.6–16.4) women living in Vapi District, representing two asymptomatic *P. falciparum* infections (Table 2). These women were primi- and secondigravidae. One of these infections was detected in the placenta. No infections were detected by RDTs.

The prevalence of maternal anaemia at delivery was 34.3 % (112/327), and the mean (95 % CI) Hb level was 11.4 (11.3–11.6) g/dL (Table 3). The mean (95 % CI) birth weight of the children was 2793 g (2745–2842 g) and 24 % of women had LBW newborns. The frequency of pre-term birth was 8.5 %. Twenty-three percent of newborns (72/313 born between 33 and 43 weeks of gestation) were small-for-gestational age according to INTERGROWTH charts. Among them, 81 % had a LBW and 19 % had a normal birth weight.

**Table 3:** Delivery and newborn characteristics, Salavan Province, Laos, 2014

	Hospital survey N = 331
	n (%) or mean (95 % CI)
Multiple birth	8/331 (2.4)
Gestational age (weeks of gestation)	38 (38–38)
Delivery outcome	
Live-birth	325 (98.2)
Stillbirth	6 (1.8)
Maternal Hb level (g/dL)^a^	11.4 (11.3–11.6)
Maternal anaemia (Hb < 11 g/dL)^a^	112/327 (34.3)
Preterm birth	28 (8.5)
Sex ratio (m/f)	0.98
Birth weight (grams)^a,b^	2793 (2745–2842)
LBW (<2500 g)^a,b^	76/317 (24.0)
SGA^a,b^	72/313 (23.0)

*Hb* haemoglobin

^a^N = 331 women at delivery or indicated otherwise. Hb level was missing for four women in Toumlane District Hospital because of a defective device

^b^Mean birth weight and proportions of LBW and SGA were calculated in live-singletons only. SGA was defined using the INTERGROWTH charts in newborns born from 33 to 43 weeks of gestation [12]

### Factors associated with low birth weight, SGA and mean birth weight

The initial statistical analysis plan included the assessment of the effect of malaria at delivery on birth weight and maternal haemoglobin level. Because of the very low prevalence of malaria at delivery, this factor could not be included in the analyses. In univariate analysis, women who smoked, were of Lao Theung ethnicity, who did not use a bed net the night before admission, who had no ANC visits and those who had a pre-term delivery were significantly more likely to have a LBW newborn (Table 4). In multivariate analysis, tobacco use (aIRR 2.43; 95 % CI 1.64–3.60) and pre-term delivery (aIRR 3.17; 95 % CI 2.19–4.57) remained significantly associated with a higher risk of LBW. In addition, women who did not use a bed net the night before admission tended to have a higher risk of LBW than those who slept under a bed net (aIRR 1.56; 95 % CI 0.92–2.64).

**Table 4:** Factors associated with low birth weight. Salavan Province, Laos, 2014

	Univariate analysis^a^	P value	Multivariate analysis^a^	P value
	Crude IRR (95 % CI)		Adjusted IRR (95 % CI)	
Age (years) (20–24 = *Ref*)		0.36		
<20	1.54 (0.92–2.60)			
25–27	1.16 (0.65–2.06)			
≥28	1.01 (0.60–1.68)			
Primigravidity	1.26 (0.85–1.87)	0.25		
Tobacco use	2.50 (1.68–3.72)	<10^−3^	2.43 (1.64–3.60)	<0.001
Ethnicity Lao Theung (*Ref* = *Lao Loum*)	1.57 (1.03–2.39)	0.04		
Place of living (*Ref* = *Salavan*)		0.17		
Vapi	0.66 (0.31–1.44)			
Toumlane	1.51 (0.93–2.44)			
Others	0.74 (0.26–2.13)			
Went to the forest during the current pregnancy	1.22 (0.83–1.82)	0.30		
No bed net use	2.07 (1.27–3.36)	0.003	1.56 (0.92–2.64)	0.10
Number of ANC visits (≥4=*Ref*)		0.01		
1–3	1.41 (0.94–2.13)			
0	2.53 (1.34–4.77)			
Gestational hypertension	1.26 (0.48–3.33)	0.64		
Moderate anaemia at delivery	1.25 (0.84–1.87)	0.28		
Duration of pregnancy (weeks gestation) (37–38 = *Ref*)		<10^−3^		<0.001
≥39	0.49 (0.25–0.99)		0.49 (0.25–0.97)	
<37	3.47 (2.51–4.81)		3.17 (2.19–4.57)	
Female	1.15 (0.77–1.70)	0.50		

*IRR* incidence rate ratio

^a^The analysis was conducted using a modified Poisson regression. Only live-singletons were included. The multivariate analysis was performed on 313 women. The final model was obtained after a backward selection procedure, bed net use was forced in the final model

SGA and mean birth weight were considered as secondary outcomes. In univariate analysis, SGA was significantly associated with the same factors as for LBW (tobacco use, bed net use, and ethnicity) (Additional file 1). In contrast, there was no association between SGA and the number of ANC visits and duration of pregnancy. In multivariate analysis, no bed net use and tobacco use remained significantly associated with a higher risk of SGA after a backward selection procedure.

When considering birth weight as a continuous variable, the same factors as for LBW as well as primigravidity and a mother’s young age (<20 years) were significantly associated with a reduction in birth weight in univariate analysis (Additional file 2). In addition, there was a marginally significant association between birth weight and gestational hypertension and visit to the forest during current pregnancy. In multivariate analysis, young age (−135 g; 95 % CI −263; −7), tobacco use (−148 g; 95 % CI −286; −10), gestational hypertension (−254 g; 95 % CI −494; −14), a low number of ANC visits (−89 g; 95 % CI −235; −33, in women with one to three visits compared to women with ≥four visits), and pre-term birth (−661 g; 95 % CI, −824; −497) remained significantly associated with a reduction in birth weight.

### Factors associated with maternal anaemia and mean haemoglobin level at delivery

In univariate analysis, women from Toumlane (compared to those from Salavan), of Lao Theung ethnicity, women who did not use a bed net the night before admission, those who made no ANC visits during pregnancy (compared to women who made ≥four ANC visits) and those who did not have iron supplementation during current pregnancy (compared to women who stated that they had iron supplementation the day before admission) were significantly more likely to be anaemic at delivery. In contrast, primigravidae and women living in Vapi District were less likely to be anaemic (Table 5). In multivariate analysis, Lao Theung ethnicity (aIRR 1.49; 95 % CI 0.99–2.25), no iron supplementation (aIRR 1.58; 95 % CI 1.08–2.32) and living in Vapi District (aIRR 0.45; 95 % CI 0.21–0.98) remained significantly associated with anaemia.

**Table 5:** Factors associated with maternal moderate anaemia. Salavan Province, Laos, 2014

	Univariate analysis^a^	P value	Multivariate analysis^a^	P value
	Crude IRR (95 % CI)		Adjusted IRR (95 % CI)	
Age (years) (20–24 = *Ref*)		0.69		
<20	1.18 (0.78–1.79)			
25–27	1.07 (0.70–1.65)			
≥28	0.90 (0.61–1.33)			
Primigravidity	0.76 (0.54–1.04)	0.10		
Tobacco use	1.31 (0.87–1.96)	0.19		
Ethnicity Lao Theung (*Ref* = *Lao Loum*)	2.07 (1.56–2.74)	<0.001	1.49 (0.99–2.25)	0.05
Place of living (*Ref* = *Salavan*)		<0.001		0.02
Vapi	0.41 (0.19–0.88)		0.45 (0.21–0.98)	
Toumlane	1.92 (1.44–2.58)		1.42 (0.94–2.16)	
Others	0.49 (0.17–1.39)		0.46 (0.16–1.33)	
Went to the forest during the current pregnancy	1.12 (0.83–1.51)	0.47		
No bed net use	1.80 (1.24–2.63)	0.002		
Number of ANC visits (≥4 = *Ref*)		0.007		
1–3	1.33 (0.98–1.82)			
0	2.09 (1.29–3.39)			
Gestational hypertension	0.79 (0.30–2.10)	0.64		
Iron supplementation (intake the day before admission = *Ref*)		0.03		0.06
Intake, but not before admission	1.02 (0.58–1.78)		1.00 (0.56–1.79)	
No intake during pregnancy	1.75 (1.16–2.62)		1.58 (1.08–2.32)	
Folic acid supplementation (intake the day before admission = *Ref*)		0.34		
Intake, but not before admission	1.25 (0.76–2.05)			
No intake during pregnancy	0.80 (0.53–1.22)			
Duration of pregnancy (weeks gestation) (37–38 = *Ref*)		0.55		
≥39	0.83 (0.56–1.24)			
<37	1.11 (0.68–1.83)			

*IRR* incidence rate ratio

^a^The analysis was conducted using a modified Poisson regression. The multivariate analysis was performed on 326 women. The final model was obtained after a backward selection procedure

When considering haemoglobin level as a continuous variable, there was a significant reduction in mean Hb level in women living in Toumlane District (compared to those living in Salavan District) and those with a low number of ANC visits (one to three compared to ≥four visits) in multivariate analysis (Additional file 3). In contrast, women living in Vapi District had a significantly higher Hb level. The association between Hb level and iron and folic acid supplementation did not remain significant after adjustment for the number of ANC visits.

## Discussion

This survey assessed the burden and epidemiology of MiP in southern Laos, where malaria transmission remains relatively high despite the large-scale distribution of both ITNs and ACT. In Vapi villages, malaria was detected by PCR in almost 6 % (12/204) of pregnant women. The majority of these infections were asymptomatic and sub-microscopic and, therefore, would not have been detected by recommended national screening strategies [7]. All 12 infections were due to *P. vivax*, among which one was mixed *P. vivax/P. falciparum*. Only two women had RDT-positive infections. PCR detected more malarial infections than RDT (frequency of malaria 5.9 vs 1.0 % using PCR vs RDT, respectively). The low proportion of RTD-positive infections is likely to be due to the low sensitivity of the test for low parasitaemia infections [13]. This may also explain the relatively large difference in malaria frequency between PCR and RDT compared to what has been reported in areas of high malaria transmission [14].

Only a single prior study had evaluated MiP in Laos, in Vientiane in 1998. This study was conducted in pregnant women with history of fever, who were recruited during ANC visits. Among 68 women, 16 (23.5 %) were diagnosed with *P. falciparum* using TBS [15]. The higher prevalence of malaria compared to the present survey may be explained by the selection of symptomatic women at a time when malaria transmission was higher. In addition, the present survey was conducted at community-level in order to reduce selection bias that arises when only those women who attend antenatal clinics are recruited.

In the villages almost 6 % of pregnant women were infected with malaria. This proportion may have been higher if longitudinal follow-up had been used, providing a cumulative proportion of infections during pregnancy. For instance, on the Thai-Burmese border, while the mean malaria prevalence during weekly cross-sectional surveys was 4 % using TBS, the cumulative proportion of women infected with malaria during pregnancy was as high as 22.8 % [3]. A history of malaria during current pregnancy was a risk factor for current malarial infections in pregnancy. These infections may correspond to a relapse of a previous *P. vivax* infection, or they may reflect a higher level of exposure to malaria of these women, compared to those who did not report previous infections.

At delivery, there was a lower prevalence of sub-microscopic infections, which varied according to antenatal clinic. In Vapi Hospital, the prevalence (4.5 %) was comparable to what had been found in the villages. However, malaria was not detected in pregnant women from Toumlane, although the sample size was very low, nor in women who delivered in Salavan Hospital, which is located in a more urban area than Vapi and where malaria transmission is probably lower. The comparison of these results with those from studies conducted in the Asia–Pacific region is difficult since malaria transmission is highly variable in this region and most of these studies included both symptomatic and asymptomatic pregnant women. On the Thai-Burmese border, where malaria transmission and women’s characteristics may be the most comparable to those in Laos, the prevalence of peripheral and placental malaria at delivery using TBS or PCR ranged from 3.0 to 10.9 % [3]. In the present study, a single sub-microscopic placental infection was detected. This may be explained by the fact that most infections (86 %) were due to *P. vivax*, which is less likely to sequester in the placenta than *P. falciparum*. In both surveys, only two out of a total of 14 infections were caused by *P. falciparum*, and *Plasmodium knowlesi* was not detected. This is in accordance with the increasing ratio between *P. vivax* and *P. falciparum* infections, which is observed in the region [3]. The higher prevalence of *P. vivax* compared to *P. falciparum* infections may also be explained by the inclusion of asymptomatic women, *P. falciparum* infections being more likely to be symptomatic.

*Plasmodium vivax* infection during pregnancy has been associated with poor maternal and child outcomes [6, 16], although its deleterious effects are lower than those observed with *P. falciparum*. A single episode of vivax or falciparum malaria can have deleterious consequences for the foetus [6, 17]. Asymptomatic and sub-microscopic infections have also been shown to be detrimental [6, 17, 18]. In this study, the direct effect of malaria on birth weight could not be assessed because of the very low number of infected women at delivery. Women who did not sleep under a bed net the night before the survey tended to have a higher risk of LBW and newborns with reduced birth weight, compared to women who used a bed net. The reduction in birth weight (−140 g) was of the same magnitude as that reported for MiP elsewhere in the region [3], making lack of bed net use a possibly proxy for exposure to malaria in this study. However, it has been shown that mosquitoes in this region have a propensity to feed outdoors and at times when women are still active and not under a bed net [19, 20]. Therefore, the association between no bed net use and a higher risk of birth weight may be explained by confounding factors, which were not taken into account in the analysis, such as a lower socio-economic status, a lower education level and worse health practices in women who did not use a bed net.

A high proportion of women (24 %) had LBW newborns, mostly due to IUGR. Indeed, newborns were classified as SGA, as a proxy for IUGR, in 23 % of cases with 80 % of LBW babies who were SGA. This is in accordance with the prevalence of LBW and the proportion of LBW due to IUGR in Asia [2]. In multivariate analysis, factors significantly associated with LBW were pre-term birth and smoking. Women who reported cigarette smoking during current pregnancy had SGA and LBW rates 2.0 and 2.4 times greater, which correspond to what has been reported elsewhere in developed countries [21]. Since 11.5 % of women reported smoking during pregnancy, the attributable risk for LBW due to smoking was as high as 14 % in this population. In addition, mothers of young age (<20 years old), a low number of ANC visits (one to three compared to ≥four visits), gestational hypertension and pre-term birth were associated with a significant reduction in birth weight (−135, −89, −254 and −661 g, respectively). These associations were found after adjustment for gravidity, which is likely correlated with these factors. Indeed, gestational hypertension and maternal age have been recognized as independent risk factors for LBW-IUGR [22, 23]. However, the association between maternal age and LBW may also have been partially confounded by maternal under-nutrition, which was not adjusted for in the present analysis. Thirty-four per cent of women were anaemic at delivery. Women who did not take iron supplementation during pregnancy had a 58 % higher risk of anaemia compared to women who stated that they took iron the day before the survey. These results highlight the importance of the prevention and treatment of gestational hypertension, smoking and iron deficiency, which are preventable risk factors for IUGR and anaemia. Women from Lao Theung ethnicity were more likely to be anaemic than women from Lao Loum ethnicity. In addition, women living in Vapi District had a lower risk for anaemia than those living in Salavan. This risk differences may be due to residual confounding, such as food habits, maternal nutritional and socio-economic status, which were not taken into account in the analysis.

The strength of the present analysis is the use of population-based data to assess the prevalence of malaria during pregnancy, limiting selection bias that arises when women are recruited at facility-level. However, the participation in the study of all eligible women in the villages cannot be warranted. Moreover, fewer women than anticipated were recruited both in the villages and at delivery, which may have led to estimating the prevalence of malaria with some inaccuracy. In Vapi and Toumlane District Hospitals all women were included at delivery, which was not the case in Salavan Provincial Hospital where a very few number of women could not be included before an emergency delivery. Finally, the high variability of malaria prevalence in south Laos limits the generalization of our results [4]. The effect of MiP on maternal anaemia and birth weight could not be assessed because of the low number of malarial infections at delivery. This will require either a higher sample size at delivery or a longitudinal follow-up of pregnant women with repeated malaria screening during pregnancy. Another weakness of this study is the limit of confounders available. Indeed, the analysis was adjusted for the available confounders, but there still remains the possibility of residual confounding. Indeed, controlling for socio-economic characteristics and maternal nutritional status may probably have attenuated the risk of LBW and anaemia with young maternal age, ethnicity, place of living and bed net use. In addition, some variables such as bed net use, history of malaria and gestational age during pregnancy were self-reported by the women and could not be verified, leading to possible misclassifications.

## Conclusions

This survey contributes a first step towards the assessment of MiP in Laos, where the prevalence of *P. vivax* sub-microscopic infections in this population was noticeable. However, these findings cannot be generalized beyond the present study area since malaria transmission is highly heterogeneous within the country [4]. Further studies in southern Laos are needed to draw a more representative picture of MiP, including both asymptomatic and sub-microscopic *Plasmodium* infections that have deleterious consequences during pregnancy and contribute to malaria transmission [13]. Systematic detection and treatment of malaria in women during ANC visits, regardless of whether they are symptomatic or not, may reduce the consequences of MiP [24]. In addition, the use of ITNs throughout pregnancy should remain a priority. Additional preventive strategies such as intermittent preventive treatment in pregnancy (IPTp) as recommended in high malaria transmission areas, may contribute to reduce the consequences of sub-microscopic infections, as well as infections which occur between ANC visits. Artemisinin-based combination therapies have been shown to be safe during pregnancy [25] and efficacious against both *P. falciparum* and *P. vivax* in such areas [26]. In particular, IPTp with dihydroartemisinin-piperaquine may be an interesting option, as recently reported in Africa [27].

## Additional files

10.1186/s12936-016-1492-2 Factors associated with small-for-gestational age (SGA). Salavan Province, Laos, 2014. Maternal characteristics associated with small-for-gestational age in both univariate and multivariate analysis using a modified Poisson regression model.
10.1186/s12936-016-1492-2 Factors associated with mean birth weight. Salavan Province, Laos, 2014. Maternal characteristics associated with mean birth weight in both univariate and multivariate analysis using a linear regression model.
10.1186/s12936-016-1492-2 Factors associated with mean haemoglobin level at delivery. Salavan Province, Laos, 2014. Maternal characteristics associated with mean haemoglobin level at delivery in both univariate and multivariate analysis using a linear regression model.

## Abbreviations

MiP: malaria in pregnancy
LBW: low birth weight
ANC: antenatal care
ITN: insecticide-treated net
IPTp: intermittent preventive treatment in pregnancy
ACT: artemisinin-based combination therapy
Hb: haemoglobin
TBS: thick blood smear
RDT: rapid diagnostic test
RT-qPCR: real-time quantitative polymerase chain reaction
IUGR: intrauterine growth retardation
IRR: incidence rate ratio
aIRR: adjusted incidence rate ratio
CI: confidence interval
IQR: interquartile range
CMPE: Centre of Malariology, Parasitology and Entomology
PALULAO: prevalence of malaria in South Lao PDR (France Expertise Internationale funded programme)
