# Supplementary material for: Prevalence of malaria in pregnancy in southern Laos: a cross-sectional survey
Source: Malar J. 2016 Aug 26;15(1):436. doi: 10.1186/s12936-016-1492-2 (PMC5002160; doi:10.1186/s12936-016-1492-2)
Supplement: Supplementary file 1 — 10.1186/s12936-016-1492-2 Factors associated with small-for-gestational age (SGA). Salavan Province, Laos, 2014. Maternal characteristics associated with small-for-gestational age in both univariate and multivariate analysis using a modified Poisson regression model. [file 12936_2016_1492_MOESM1_ESM.docx]

**Additional file 1.**

|  | Univariate analysis^*^ |  | Multivariate analysis^*^ |  |
| --- | --- | --- | --- | --- |
|  | Crude IRR  (95% CI) | P value | Adjusted IRR  (95% CI) | P value |
| Age (years) *(20-24=Ref)* |  | 0.27 |  |  |
| <20 | 2.11 (0.98-4.55) |  |  |  |
| 25-27 | 1.58 (0.73-3.38) |  |  |  |
| ≥28 | 1.35 (0.69-2.64) |  |  |  |
| Primigravidity | 1.24 (0.73-2.12) | 0.43 |  |  |
| Tobacco use | 3.07 (1.47-6.42) | 0.003 | 2.04 (1.29-3.24) | 0.002 |
| Ethnicity Lao Theung (*Ref=Lao Loum*) | 1.86 (0.99-3.47) | 0.05 |  |  |
| Place of living (*Ref=Salavan*) |  | 0.24 |  |  |
| Vapi | 1.17 (0.52-2.63) |  |  |  |
| Toumlane | 2.20 (1.03-4.68) |  |  |  |
| Others | 1.50 (0.51-4.41) |  |  |  |
| Went to the forest during the current pregnancy | 1.23 (0.73-2.10) | 0.44 |  |  |
| No bed net use | 2.50 (1.02-6.13) | 0.04 | 1.69 (0.94-3.01) | 0.08 |
| Number of ANC visits *(*≥*4=Ref)* |  | 0.58 |  |  |
| 1-3 | 1.20 (0.69-2.08) |  |  |  |
| 0 | 1.83 (0.53-6.41) |  |  |  |
| Gestational hypertension | 2.31 (0.63-8.40) | 0.21 |  |  |
| Moderate anaemia at delivery | 0.96 (0.55-1.70) | 0.90 |  |  |
| Duration of pregnancy (weeks gestation) *(37-38=Ref)* |  | 0.87 |  |  |
| ≥39 | 0.91 (0.48-1.71) |  |  |  |
| <37 | 0.74 (0.20-2.71) |  |  |  |
| Female | 1.07 (0.63-1.80) | 0.81 |  |  |

^*^ The analysis was conducted using a modified Poisson regression. SGA was defined according to INTERGROWTH charts [12]. Only live-singletons born between 33 and 43 weeks of gestation were included. The multivariate analysis was performed on 313 women. The final model was obtained after a backward selection procedure, bed net use was forced in the final model.

IRR: incidence rate ratio.
