# Supplementary material for: Prevalence of malaria in pregnancy in southern Laos: a cross-sectional survey
Source: Malar J. 2016 Aug 26;15(1):436. doi: 10.1186/s12936-016-1492-2 (PMC5002160; doi:10.1186/s12936-016-1492-2)
Supplement: Supplementary file 2 — 10.1186/s12936-016-1492-2 Factors associated with mean birth weight. Salavan Province, Laos, 2014. Maternal characteristics associated with mean birth weight in both univariate and multivariate analysis using a linear regression model. [file 12936_2016_1492_MOESM2_ESM.doc]

**Additional file 2.**

|  | Univariate analysis |  | Multivariate analysis^*^ |  |
| --- | --- | --- | --- | --- |
|  | Crude coefficient  (95% CI) | P value | Adjusted coefficient  (95% CI) | P value |
| Age (years) *(20-24=Ref)* |  | 0.13 |  |  |
| <20 | -160 (-306 ; -14) |  | -135 (-263 ; -7) | 0.04 |
| 25-27 | -7 (-149 ; 134) |  | -45 (-170 ; 79) | 0.48 |
| ≥28 | 11 (-107 ; 129) |  | -28 (-131 ; 75) | 0.60 |
| Primigravidity | -113 (-212 ;-13) | 0.03 |  |  |
| Tobacco use | -170 (-324 ; -16) | 0.03 | -148 (-286 ; -10) | 0.03 |
| Ethnicity Lao Theung (*Ref=Lao Loum*) | -144 (-267; -20) | 0.02 |  |  |
| Place of living (*Ref=Salavan*) |  | 0.34 |  |  |
| Vapi | 55 (-96; 207) |  |  |  |
| Toumlane | -118 (-267; 32) |  |  |  |
| Others | 22 (-196; 239) |  |  |  |
| Went to the forest during the current pregnancy | -87 (-185; 11) | 0.08 |  |  |
| No bed net use | -228 (-410; -46) | 0.01 | -140 (-300; 20) | 0.08 |
| Number of ANC visits *(*≥*4=Ref)* |  | 0.02 |  |  |
| 1-3 | -134 (-235 ; -33) |  | -89 (-235 ; -33) | 0.05 |
| 0 | -150 (-405 ; 105) |  | 59 (-168 ; 286) | 0.61 |
| Gestational hypertension | -234 (-511; 43) | 0.10 | -254 (-494; -14) | 0.04 |
| Moderate anaemia at delivery | -47 (-149; 56) | 0.37 |  |  |
| Duration of pregnancy (weeks gestation) *(37-38=Ref)* |  | <10^-3^ |  | <0.001 |
| ≥39 | 224 (121 ;-326) |  | 219 (117 ; 321) |  |
| <37 | -679 (-845 ;-513) |  | -661 (-824 ; -497) |  |
| Female | -15 (-112 ; 82) | 0.76 |  |  |
| Constant |  |  | 2,862 (2,787 ; 2,937) |  |

^*^ Only live-singletons have been included. The multivariate analysis was performed on 313 women. The final model was obtained after a backward selection procedure, bed net use was forced in the final model.
