# Supplementary material for: Prevalence of malaria in pregnancy in southern Laos: a cross-sectional survey
Source: Malar J. 2016 Aug 26;15(1):436. doi: 10.1186/s12936-016-1492-2 (PMC5002160; doi:10.1186/s12936-016-1492-2)
Supplement: Supplementary file 3 — 10.1186/s12936-016-1492-2 Factors associated with mean haemoglobin level at delivery. Salavan Province, Laos, 2014. Maternal characteristics associated with mean haemoglobin level at delivery in both univariate and multivariate analysis using a linear regression model. [file 12936_2016_1492_MOESM3_ESM.docx]

**Additional file 3.**

|  | Univariate analysis |  | Multivariate analysis^*^ |  |
| --- | --- | --- | --- | --- |
|  | Crude coefficient  (95% CI) | P value | Adjusted coefficient  (95% CI) | P value |
| Age (years) *(20-24=Ref)* |  | 0.86 |  |  |
| <20 | -0.18 (-0.69 ; 0.32) |  |  |  |
| 25-27 | 0.05 (-0.44 ; 0.54) |  |  |  |
| ≥28 | 0.02 (-0.39 ; 0.43) |  |  |  |
| Primigravidity | -0.32 (-0.02 ; 0.67) | 0.07 |  |  |
| Tobacco use | -0.29 (-0.82 ; 0.24) | 0.29 |  |  |
| Ethnicity Lao Theung (*Ref=Lao Loum*) | -0.98 (-1.40; -0.56) | <10^-3^ |  |  |
| Place of living (*Ref=Salavan*) |  | <10^-3^ |  | <10^-3^ |
| Vapi | 0.71 (0.22; 1.19) |  | 0.92 (0.46; 1.39) |  |
| Toumlane | -1.04 (-1.55; -0.53) |  | -0.95 (-1.42; -0.47) |  |
| Others | 0.43 (-0.29; 1.14) |  | 0.49 (-0.17; 1.15) |  |
| Went to the forest during the current pregnancy | -0.10 (-0.44; 0.24) | 0.57 |  |  |
| No bed net use | -1.00 (-1.64; -0.37) | 0.002 | - |  |
| Number of ANC visits *(*≥*4=Ref)* |  | 0.03 |  | 0.004 |
| 1-3 | -0.50 (-0.85 ; -0.15) |  | -0.50 (-0.82 ; -0.18) |  |
| 0 | -0.72 (-1.58 ; 0.14) |  | -0.67 (-1.44 ; 0.11) |  |
| Gestational hypertension | -0.18 (-1.11; 0.76) | 0.71 |  |  |
| Iron supplementation  (Intake the day before admission *=Ref*) |  | 0.26 |  |  |
| Intake, but not before  admission | -0.15 (-0.76 ; 0.47) |  |  |  |
| No intake during  pregnancy | -0.56 (-1.25 ; 0.13) |  |  |  |
| Folic acid supplementation  (Intake the day before admission*=Ref*) |  | 0.03 |  |  |
| Intake, but not before  admission | -0.43 (-1.05; 0.19) |  |  |  |
| No intake during  pregnancy | 0.42 (0.03 ; 0.81) |  |  |  |
| Duration of pregnancy (weeks gestation) *(37-38=Ref)* |  | 0.82 |  |  |
| ≥39 | 0.10 (-0.31 ; 0.51) |  |  |  |
| <37 | -0.10 (-0.71 ; 0.51) |  |  |  |
| Constant |  |  | 11.59 (11.38 ; 11.81) |  |

^*^ The multivariate analysis was performed on 322 women. The final model was obtained after a backward selection procedure, bed net use was forced in the final model.
